# Mechanical Impedance of the Non-loaded Lower Leg with Relaxed Muscles in the Transverse Plane

**DOI:** 10.3389/fbioe.2015.00198

**Published:** 2015-12-08

**Authors:** Evandro Maicon Ficanha, Guilherme Aramizo Ribeiro, Mohammad Rastgaar

**Affiliations:** ^1^HIRoLab, Department of Mechanical Engineering-Engineering Mechanics, Michigan Technological University, Houghton, MI, USA

**Keywords:** lower leg impedance, lower leg stiffness, impedance estimation, lower leg external–internal rotations, lower leg transverse plane

## Abstract

This paper describes the protocols and results of the experiments for the estimation of the mechanical impedance of the humans’ lower leg in the External–Internal direction in the transverse plane under non-load bearing condition and with relaxed muscles. The objectives of the estimation of the lower leg’s mechanical impedance are to facilitate the design of passive and active prostheses with mechanical characteristics similar to the humans’ lower leg, and to define a reference that can be compared to the values from the patients suffering from spasticity. The experiments were performed with 10 unimpaired male subjects using a lower extremity rehabilitation robot (Anklebot, Interactive Motion Technologies, Inc.) capable of applying torque perturbations to the foot. The subjects were in a seated position, and the Anklebot recorded the applied torques and the resulting angular movement of the lower leg. In this configuration, the recorded dynamics are due mainly to the rotations of the ankle’s talocrural and the subtalar joints, and any contribution of the tibiofibular joints and knee joint. The dynamic mechanical impedance of the lower leg was estimated in the frequency domain with an average coherence of 0.92 within the frequency range of 0–30 Hz, showing a linear correlation between the displacement and the torques within this frequency range under the conditions of the experiment. The mean magnitude of the stiffness of the lower leg (the impedance magnitude averaged in the range of 0–1 Hz) was determined as 4.9 ± 0.74 Nm/rad. The direct estimation of the quasi-static stiffness of the lower leg results in the mean value of 5.8 ± 0.81 Nm/rad. An analysis of variance shows that the estimated values for the stiffness from the two experiments are not statistically different.

## Introduction

The ankle is the first major joint that transfers the ground reaction forces to the rest of the body during walking. Reaction forces allow for propulsion and the changing of direction of gait. Activities of daily leaving (ADLs) involve different tasks requiring the lower leg to function in all the anatomical planes. For example, during normal walking, walking on inclined planes, turning around corners, avoiding obstacles, and climbing/descending stairs, the lower leg dynamics change continuously to accommodate for these different maneuvers and conditions of agile gait. Studies of four representative daily activities show that, depending on the activity, turning steps may account for an average of 25% (range 8–50%) of all daily steps (Glaister et al., 2007a). Therefore, the development of ankle–foot prostheses should take the mechanical characteristics of the human lower leg into consideration.

Currently, available powered ankle–foot prostheses focus on improving mobility by powering the ankle joint in the sagittal plane; however, there is substantial ankle function in all anatomical planes, even during straight walk on level ground (Weyand et al., 2000; Taylor et al., 2005; Ficanha et al., 2015b). Ankle–foot prostheses with anthropomorphic characteristics may improve the metabolic cost while generating a more comfortable gait and decreasing the secondary injuries due to overuse or misuse of other joints. These may increase mobility and activity levels, reduce the likelihood of obesity and cardiovascular diseases, and overall improve the quality of life in amputees.

The ankle and lower leg kinetics and kinematics show significant variability when comparing the angles and torques during straight walking and sidesteps cutting (a step where the leading leg pushes the body sideways near or at 45° to avoid an obstacle on the ground while walking forward) in both external–internal (EI) and inversion–eversion (IE). It has been shown that during a sidestep cutting maneuver at normal walking speed, the ankle torque in the lateral direction at the push off phase increased more than six times compared to walking on a straight path (Ficanha et al., 2015b). This result indicated that a torque in the transverse plane is transferred from the human body to the ground through the ankle during walking on a straight path. The amount of transferred torque is larger during turning. On the other hand, the ankle angles during these two walking scenarios show no statistical differences, which indicates that a higher stiffness of the ankle is required for turning maneuvers. This higher stiffness is necessary to transfer the torque from the hip joint to the ground, so the ground reaction torques would steer the body into the new walking direction. The ankle is capable of considerable movement in the transverse plane, allowing the body to rotate in the transverse plane while the foot remains in contact with the floor (Nester et al., 2003). This evidence suggests that the lower leg dynamics and its variable stiffness may play a significant role during walking and turning when the lower leg’s muscles undergo co-contraction.

The ankle is composed of the talocrural and the subtalar joints, which are not aligned with the anatomical axes. The rotation of the ankle about each of these joints results in combined rotations in the anatomical reference frame. The talocrural joint combines dorsiflexion with lateral rotation and also combines plantarflexion with medial rotation. The subtalar joint combines dorsiflexion, eversion, and external rotation and, additionally, it combines plantarflexion, inversion, and internal rotation (Wheeless, 2012). The tibia and the talus of the foot form the talocrural joint, and the tibia is in contact with the fibula at their lower and upper ends as the tibiofibular joints. The movements of the tibiofibular joints are small and usually neglected in gait analysis (Levine et al., 2012). In the presented experiment, however, the kinematics and kinetics, hence the impedance of the lower leg in the transverse plane is presented as a combination of the talocrural and subtalar joints’ functions and any contribution from the tibiofibular and knee joints. During the experiments presented in this paper, the angular movement of the lower leg in the global reference frame and the applied torque by the Anklebot were recorded. Therefore, the estimated impedance is the lower leg rotational impedance in the transverse plane contributed by the ankle and lower leg musculoskeletal system. The stiffness of the leg in the transverse plane is also affected by the angle of the knee joint; when the leg is stretched, the hip rotation in the transverse plane is present at the lower leg and changes the impedance measurement. Since the tests were performed with the knee at a 90° angle, the effects of the hip joint on the results were not present. Therefore, the results are merely valid for the conditions of the performed experiments.

The mechanical impedance of a system is defined as the evoked torque due to input motion perturbations, and it is a function of the systems mass, damping, and stiffness. The ankle impedance in the frontal and sagittal planes have been previously studied with relaxed and co-contracting muscles under no-load condition using single and multivariable stochastic identification approaches (Hunter and Kearney, 1982; Kearney and Hunter, 1982; Weiss et al., 1986a,b; Kirsch and Kearney, 1997; Lortie and Kearney, 2001; Ludvig et al., 2001; Rastgaar et al., 2009, 2010; Zhao et al., 2010; Lee et al., 2011a, 2014b; Ficanha and Rastgaar, 2014). The ankle impedance variation in the sagittal plane during the foot-flat sub-phase of stance was estimated using a perturbation platform (Rouse et al., 2014). Additionally, the time-varying dynamic mechanical impedance of the ankle in sagittal and frontal planes during pre-swing, swing, and early stance of the gait was studied using the Anklebot (Lee and Hogan, 2014). Both those studies showed consistent time-varying behavior of the ankle mechanical impedance during gait.

Powered lower extremity prostheses usually have one degree of freedom (DOF) in the sagittal plane (Sup et al., 2009; Eilenberg et al., 2010; Hitt et al., 2010). The authors have developed a 2-DOF powered ankle–foot prosthesis with two controllable DOFs in the sagittal and frontal planes (Ficanha et al., 2014, 2015c). The design of powered ankle–foot prostheses generally considers the lower leg and ankle to be fixed in the transverse plane, since the hip joint is capable of generating the majority of torques that is transferred to the ground for steering and turning. Nevertheless, the ankle and lower leg rotate in the transverse plane during the gait and should be designed as a compliant joint in this anatomical plane. Recently, Olson et al. developed a transtibial prosthesis with active control in the transverse plane (Olson and Klute, 2015). This device is capable of generating torque in single DOF in the transverse plane and uses an impedance controller. A better understanding of the impedance of the lower leg in the transverse plane may lead to the development of ankle–foot prostheses that can properly mimic the mechanical characteristics of the human lower leg in EI (Glaister et al., 2009). Compliance in the transverse plane may reduce the painful shear stresses on the residual limbs (Glaister et al., 2007b). Variable stiffness mechanisms could be developed to mimic the time-varying impedance of the ankle and lower leg, facilitating ADLs, reducing secondary injuries, and improving overall quality of life for lower extremity amputees.

This paper presents the methods and the results of the estimation of the dynamic mechanical impedance and quasi-static stiffness of the human lower leg under no-load bearing condition and with relaxed muscles in the transverse plane. This study is aimed to improve our understanding of the dynamics of the lower leg function by developing experiments that may lead to better explaining how its muscle activation may modulate its mechanical impedance in the transverse plane. The practical benefits of this information may lead to characterizing the primary design parameters for the development of the ankle–foot prostheses with anthropomorphic characteristics. Additionally, the results can be used as a reference for comparing the results from patients suffering from spasticity due to stroke or patients with multiple sclerosis. Lee et al. (2011b) compared the results of the quasi-static stiffness of the ankle in individual suffering from stroke and multiple sclerosis with unimpaired subjects. They showed some quantitative differences in the results of the mechanical impedance of the ankle in dorsiflexion–plantarflexion (DP) and IE. An impedance estimation experiment may be used to examine the patients’ level of impairment, or to monitor their progress during treatment.

In this paper, the experiments were performed using a lower extremity rehabilitation robot on 10 unimpaired male subjects. In the quasi-static test, the robot applied ramp perturbations at 0.4 rad/sec to the lower leg and the resultant lower leg torque was recorded. In the dynamic impedance estimation experiment, the robot applied random torque perturbations to the lower leg and a stochastic identification method was used. The stochastic identification method was used since it does not require any *a priori* information about the dynamics of the system, making it suitable for analysis of complex mechanical systems such as the human lower leg. The stochastic method presented has the advantage of applying equal amounts of energy at all frequencies within the studied frequency range. The paper first describes the experiment setup to use the Anklebot to apply perturbations to the lower leg. Next, the experiment protocol and results for dynamic impedance estimation and quasi-static stiffness estimation are presented and discussed.

## Experiment Methodology

### Human Subjects

Ten male subjects with no self-reported neuromuscular and biomechanical disorders were recruited for the experiments (ages ranging from 23 to 28 years and body mass index (BMI) ranging from 22.4 to 30.0). The subjects gave written consent to participate in the experiment, which was approved by the Michigan Tech Institutional Review Board.

### Experimental Setup

**Figure 1:**
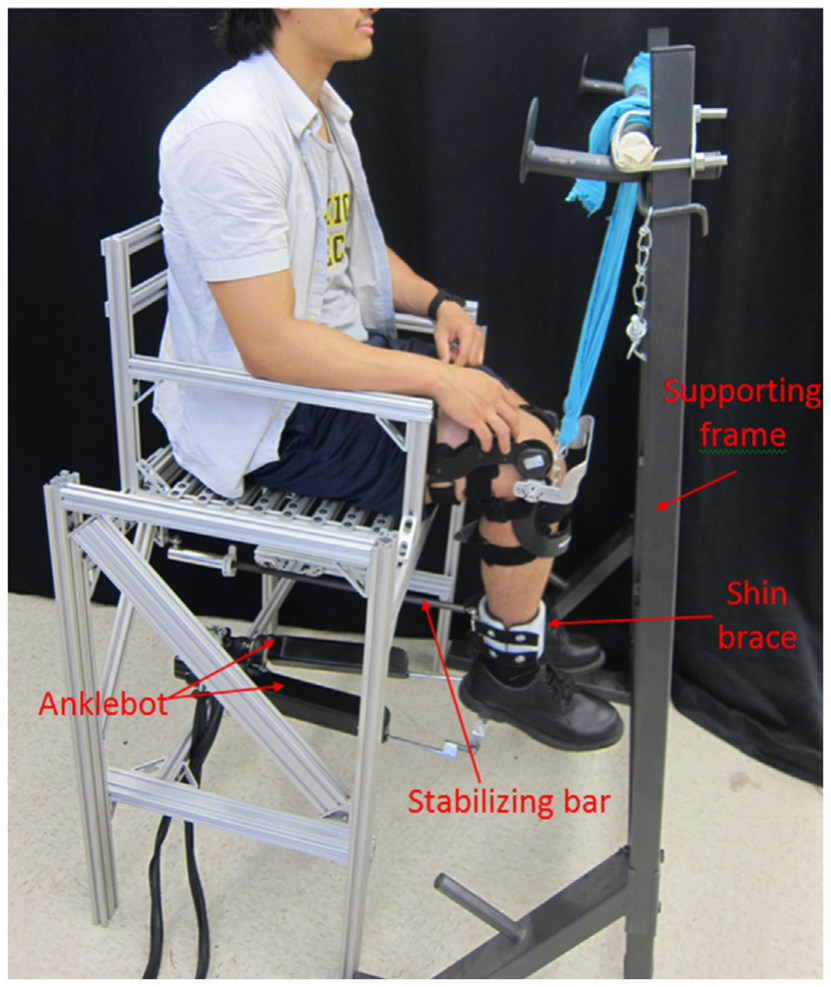
**Test setup for the estimation of the dynamic mechanical impedance and the quasi-static stiffness of the human lower leg in external–internal rotation direction**.

A wearable lower extremity rehabilitation robot capable of applying controlled torque perturbations to the lower leg, Anklebot, was used to apply torque perturbations in EI direction. The Anklebot records the applied torques and the angular displacement of the lower leg as the result of the applied perturbations as described in detail by Roy et al. (2009). The Anklebot is backdrivable with low friction; therefore, it allows the users to move their foot relative to the shank. It consists of two nearly parallel linear actuators attached to the leg (through a shoe) as seen on Figure 1. Position information is provided by two Renishaw linear incremental encoders with a resolution of 5 × 10^−6^ m mounted on the traction drives. Torque is measured by current sensors (Burr-Brown 1NA117P), which provide a measure of motor torque with a nominal resolution of 2.59 × 10^−6^ Nm.

The Anklebot has been previously used for estimation of the ankle mechanical impedance in both DP and IE (Lee et al., 2009, 2010, 2011a, 2014a,b,c; Rastgaar et al., 2009, 2010; Ficanha and Rastgaar, 2014). In these studies, the actuators were placed parallel to the shin and aligned approximately between the knee and the ball of the foot. The Anklebot was attached to the leg through a knee brace and a modified shoe. The weight of the Anklebot was supported by mounting the knee brace to the chair or by hanging on a horizontal bar. In this configuration, the sum of the actuator forces generated a DP torque, and their difference generated an IE torque. The maximum capacity of the Anklebot in applying controllable torques in DP is 23 Nm and in IE is 15 Nm, simultaneously.

For the estimation of the mechanical impedance of the lower leg in EI direction, the configuration of the Anklebot was modified, allowing its actuators to apply forces in the transverse plane. As shown in Figure 1, a testing chair was fabricated and the Anklebot was securely mounted to the chair horizontally. One end of each actuator was mounted to a horizontal bar that was fixed between the two rear legs of the chair. The moving ends of the actuators were mounted to an aluminum bracket of a modified shoe that was worn by the human subjects. The shoe allowed the force applied by the actuators to rotate the foot in the transverse plane. A stabilizing bar and shin brace were used to constrain the leg from swinging. At one end, the stabilizing bar was mounted underneath the chair using a spherical joint, and at the other end it was connected using a second spherical joint to a semi-cylinder Polyethylene component. The spherical joints allow the shin to rotate in all the anatomical planes, while constraining the translation of the leg in the sagittal plane. A second semi-cylinder Polyethylene component was connected to the first Polyethylene component using two lashing straps forming the shin brace. The lashing straps allow the shin brace to be adjusted to the users’ leg dimensions, and the shin brace connects the stabilizing bar to the leg. The internal parts of the shin brace were padded with rubber foam to increase comfort and avoid slippage against the user’s skin. A supporting frame was used to support the weight of the Anklebot, shoe, and subject’s leg through a knee brace, and to keep the Anklebot actuator at a 90° angle with respect to the shin in the sagittal plane. At the beginning of each test, the foot was centered at a neutral position using a goniometer. At that position, the knee brace height with respect to the supporting frame was set so the shoe brackets were at the same height as the Anklebot actuators; assuring the Anklebot actuators were at a 90° angle with respect to the shin in the sagittal plane. Next, the shin brace was attached to the user’s shin, and the Anklebot was attached to the shoe. The Anklebot position encoders were set to zero, making this position the reference point for the Anklebot. The actuators of the Anklebot moved the same amount during the tests, but in opposite directions in the sagittal plane. The shin brace constrained the shin from translating in the sagittal plane. This way, the lower leg center of rotation remained in the same place. In this configuration, the actuators generated EI rotations of the foot by providing input displacements with identical magnitude and opposite direction with a maximum torque of 15 Nm.

**Figure 2:**
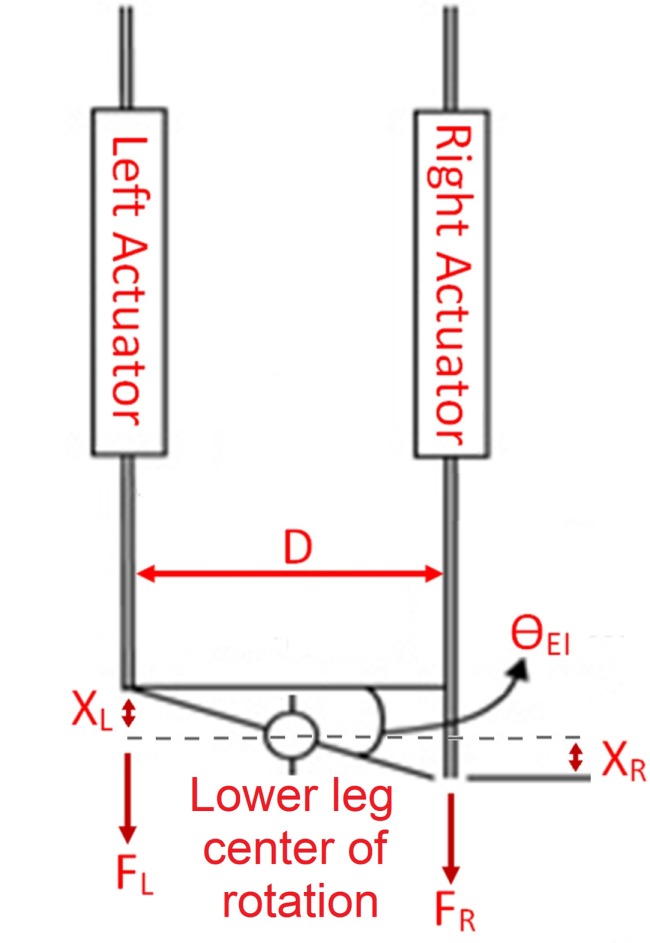
**Schematics of the Anklebot during the experiments**. The actuators displacements X_L_ and X_R_ are equal in magnitude and opposite in direction. The lower leg is constrained from translation in the same direction as the displacements X_L_ and X_R_, thus, the lower leg center of rotation does not move.

Figure 2 shows the schematic of the Anklebot during the experiments. The measured variables were the linear displacement of the left and right actuators of the Anklebot (X_L_ and X_R_, respectively) and the actuation force of each actuator (F_L_ and F_R_, respectively). To calculate the lower leg impedance and the quasi-static stiffness, both the angles and torques were required. Equations 1 and 2 were used to calculate the angle of the foot (θ*_*EI*_*) and applied torques (τ*_*EI*_*) in the EI direction based on the kinematics model of the experiment setup shown in Figure 2, where D is the distance between the actuators and was set at 0.16 m.

(1) $$\tau_{\mathrm{EI}}=\frac{(F_{R}-F_{L})\times D}{2}$$

(2) $$\theta_{\mathrm{EI}}=\arctan(\frac{X_{L}-X_{R}}{D})$$

### Dynamic Impedance Estimation

During the experiments, the subjects were instructed to remain relaxed with no muscle contraction. The Anklebot was set to generate pseudo-random voltage inputs with a bandwidth of 100 Hz. The voltage inputs to the actuators were similar in magnitude but with opposite signs, generating lower leg rotations in EI. The ankle range of motion in EI during straight walking at normal speed is near 0.26 rad (Ficanha et al., 2015b). The perturbations were set to generate the angular displacement of the lower leg with a root-mean-squared (rms) value of 0.065 rad to assure that the resulting angular displacements were within a linear range of motion. The Anklebot applied the perturbations for 70 s and recorded the data for the applied force and displacement of the actuators at 200 Hz. The first 5 s of the recorded data were discarded to remove the effects of any transient interaction dynamics and the initial adaptation of the participants. Next, the angles of rotation of the lower leg and the applied torques were calculated as described in Eqs 1 and 2.

The Anklebot generated random torque inputs to the foot, which resulted in the rotation of the lower leg in the transverse plane. This system is properly defined as a mechanical admittance that admits torque inputs (τ) and generates motion output (θ). Linear dynamics were assumed based on small angular displacements of the applied perturbations (0.065 rad rms) with respect to the range of motion of the ankle in the EI (near 0.26 rad) during normal walking (Ficanha et al., 2015b). Assuming linear dynamics, the admittance *Y* (as a function of frequency *f*), is the transfer function:

(3) θ=Y(f)τ

The impedance *Z*(*f*) is defined as the inverse of the admittance:

(4) $$Z\left(f\right)=Y^{-1}\left(f\right)$$

In the experiments, the impedance function correlates the input angles to the output torques in EI:

(5) $$\tau_{\mathrm{EI}}=Z(f)\theta_{\mathrm{EI}}$$

During the experiments, a proportional controller with gain *K* = 10 Nm was added to the Anklebot controller to hold the foot near its central position and avoid its drift. This value was determined by trial and error to properly hold the foot in its central position for all the users during the experiments. Therefore, the total measured torque τ is the sum of the proportional controller torque and the human torque:

(6) $$\tau =k(\theta_{o}-\theta_{\mathrm{EI}})+\tau_{\mathrm{EI}}$$

where θ*_*o*_* is the lower leg angle in the neutral position. In the beginning of each test, the foot is centered and the encoders are set to 0, resulting in the neutral position θ*_*o*_* to be always 0. Combining and simplifying Eqs 3 and 4 results in:

(7) $$\tau (f)=\left(Z\left(f\right)+k\right)\theta_{\mathrm{EI}}(f)$$

Solving for *Z*(*f*) in Eq. 5 yields:

(8) $$Z(f)=\frac{\tau \left(f\right)}{\theta_{\mathrm{EI}}\left(f\right)}-k$$

where τ(*f*) and θ*_*EI*_*(*f*) are the torque and angle measurements from the experiment. To calculate the impedance function, the Matlab’s^®^ built in function *tfestimate* was used. The function *tfestimate* finds a transfer function based on the quotient of the cross power spectral density of the torques and angles and the auto power spectral density of the torques. The *tfestimate* was used with a Hamming window of 512 samples, 50% overlap, and evaluated with a fast Fourier transform length of 1024 samples, resulting in a spectral resolution of 0.19 Hz. The coherence between the input angle and output torque was calculated with the Matlab’s^®^ function *mscohere* with the same parameters as the *tfestimate* function. The *mscohere* is a function of frequency with values between 0 and 1 that indicates how well the input correlates to the output at each frequency. The coherence is a function of the power spectral density of the angles, the power spectral density of the torques, and the cross power spectral density of the angles and torques. A final step was required to separate the Anklebot dynamics from the subject’s lower leg dynamics. The Anklebot, shoe, and lower leg share the same motion, while the torque measurement is the sum of the torques required to move all of them together. As a result, the estimated dynamic impedance in Eq. (6) is the sum of the impedances of the lower leg, shoe, and Anklebot. A similar experiment with no human participation was conducted to estimate the impedance of the Anklebot and shoe together. The human impedance Z*_*ankle*_* is the difference between the estimated impedance functions resulted from the two experiments with the human participants (Z*_*lower leg*_*_+_*_*Anklebot*_*_+_*_*shoe*_*) and without the human participants (Z*_*Anklebot*_*_+_*_*shoe*_*):

(9) $${\text{Z}}_{\mathrm{ankle}}={\text{Z}}_{\mathrm{lower}\mathrm{leg}}{\;}_{+\mathrm{Anklebot}+\mathrm{shoe}}-{\text{Z}}_{\mathrm{Anklebot}}{\;}_{+\mathrm{shoe}}$$

### Quasi-Static Stiffness Estimation

The experiments for the estimation of the quasi-static stiffness were performed with the same setup used for the estimation of the dynamic impedance of the lower leg. The quasi-static experiments were performed to validate the results obtained from the impedance estimation experiments. During the quasi-static experiments, the Anklebot was operated in a position control mode with a stiffness of 2177 Nm/rad and damping of 100 Nms/rad. These values were found experimentally to generate smooth operation of the Anklebot. Each experiment consisted of rotating the lower leg for 0.4 rad (from the central position) in the internal direction followed by a 0.4 rad rotation in the external direction and back to the central position with a constant speed of 0.4 rad/s. This cycle was repeated 10 times without any pause. Below the break frequencies of the estimated dynamic impedance, the visco-elastic elements play the dominant role. The quasi-static experiment is focused on the dynamics of the lower leg in very low frequencies; thus, the speed of the quasi-static experiments should be low. However, very low speed would require long experimental time and caused non-linearities due to stiction in the Anklebot actuators. The loading speed of 0.4 rad/s was selected experimentally, as it is was found to minimize the effects of the Anklebot’s actuator stiction for a smooth operation. The loading speed of 0.4 rad/s is equivalent to 0.06 Hz, or about 1.74% of the break frequency (which was found to be 4.4 ± 0.22 Hz, as it will be described later in the paper).

The recorded angles and torques were filtered with a low pass filter with a cutoff frequency of 1 Hz. For each subject, the lower leg crossed the reference angle (0 degrees) 10 times. To estimate the slopes of each segment (the quasi-static stiffness of the lower leg in Nm/rad), a second-order polynomial was fit to each segment in a least square sense. The 10 segments obtained from each test subject were averaged to obtain a representative quasi-static stiffness value for that subject.

## Results

### Results for Dynamic Impedance

**Figure 3:**
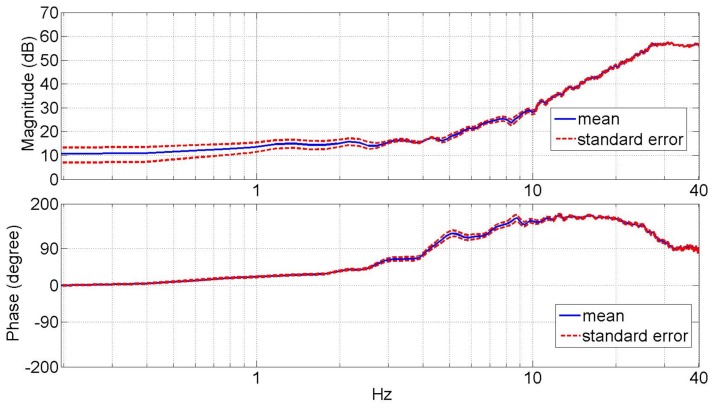
**Average magnitude and phase plots of the lower leg dynamic mechanical impedance in the external–internal rotation direction**.

**Figure 4:**
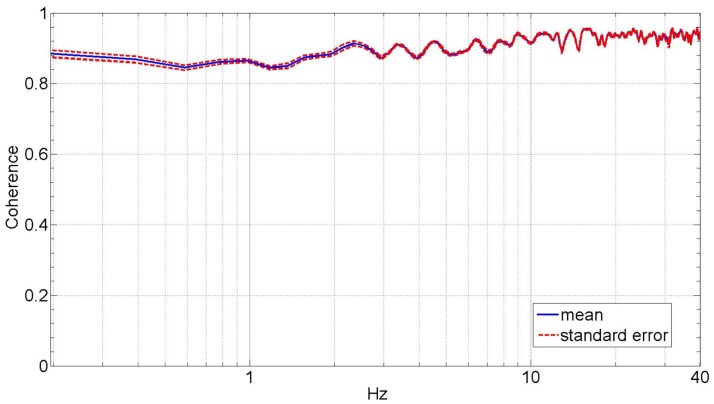
**Average coherence plot of the lower leg dynamic mechanical impedance in the external–internal rotation direction**.

Figure 3 shows the average dynamic impedance of the lower leg across the subjects as a frequency response plot within the frequency range of up to 30 Hz. The top plot shows the magnitude and the bottom plot shows the phase of the dynamic impedance. The average break frequency, where the phase plot crosses 90°, was found to be 4.4 ± 0.22 Hz. The average magnitude plot shows a slope of 46.6 ± 1.5 dB/decade beyond the break frequency. The average magnitude below the break frequency was 6.0 ± 0.85 Nm/rad (15.6 ± 1.4dB). Figure 4 shows the coherence plot for the dynamic impedance function. The average coherence in the range of 0–30 Hz was 0.92 ± 0.004, with a minimum of 0.85 at 0.89 Hz. The high value of the coherence validates the assumption of the linearity of the dynamic impedance within this frequency range and the conditions of the experiment.

At very low frequencies (0–1 Hz), the magnitude plot of the dynamic impedance provides information about the quasi-static stiffness of the lower leg. The quasi-static stiffness magnitude for each test subject averaged over 0–1 Hz is shown in Table 1. The average quasi-static stiffness magnitude across the human subjects was 4.90 Nm/rad (13.8 dB), with a SE of 0.74 Nm/rad (2.6 dB). A second-order system was fit to the data using the MATLAB^®^ function *tfest* for the impedance results of each subject. The *tfest* function estimates a transfer function from frequency domain data using the prediction error minimization approach to estimate the transfer function’s coefficients. From the second-order model, the inertia, damping, and stiffness of each subject’s lower leg were estimated as shown in Table 2. The average stiffness from the transfer functions was 4.66 Nm/rad (13.37 dB) with a SE of 0.70 Nm/rad (3.01 dB). The average damping was 2.25 ± 0.43 Nm/(rad/s) and the average moment of inertia was 0.37 ± 0.03 kg.m^2^. The average dynamic impedance transfer function was determined as Z(s) = 0.37/(s^2^ + 6.08s + 12.6).

**Table 1:** **Quasi-static stiffness estimated from the averaged impedance in the range of 0–1 Hz of the participants’ lower leg in EI direction averaged in the range of 0–1 Hz**.

Subject number	Average impedance magnitude in the range of 0–1 Hz (Nm/rad)
1	7.82
2	4.51
3	5.64
4	3.52
5	3.99
6	3.03
7	4.86
8	3.07
9	2.63
10	9.95
Mean	4.90
SE	0.74

**Table 2:** **Inertia, damping, and stiffness estimated from the dynamic impedance magnitude and phase of the participants’ lower leg in EI direction in the range of 0–30 Hz**.

Subject number	Estimated stiffness (Nm/rad)	Estimated damping Nm/(rad/s)	Estimated inertia (kg m^2^)
1	6.85	0.95	0.30
2	5.39	0.80	0.26
3	5.73	3.54	0.44
4	3.38	2.14	0.33
5	3.87	2.23	0.43
6	2.72	1.83	0.32
7	4.74	2.44	0.52
8	2.96	1.93	0.35
9	1.80	1.19	0.25
10	9.15	5.40	0.51
Mean	4.66	2.25	0.37
SE	0.03	0.43	0.70

### Results for Direct Estimation of the Quasi-Static Stiffness

**Figure 5:**
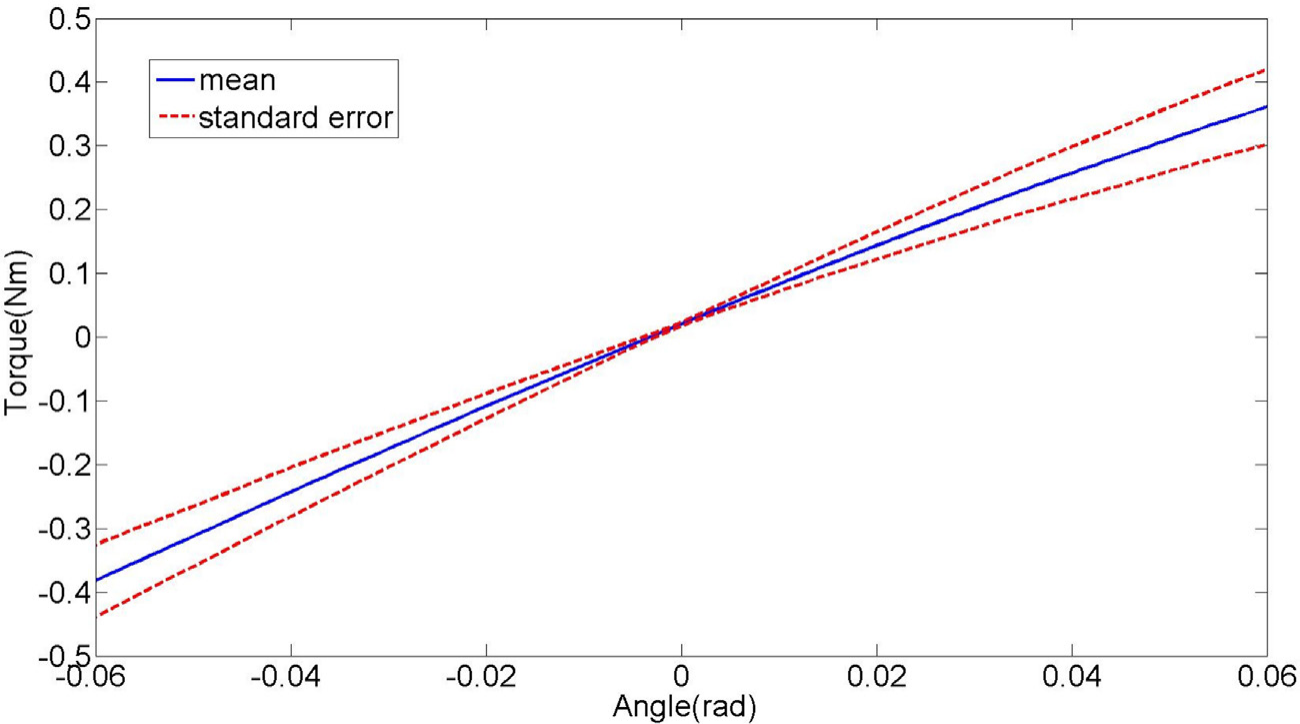
**Plot of the average lower leg torque vs. lower leg angle during the quasi-static stiffness experiment in external–internal rotation direction**. The slope represents the stiffness of the lower leg.

The quasi-static stiffness of the lower leg in EI was estimated directly for each subject and the results are shown in Table 3. The average torques applied to the foot of all the subjects plotted against the corresponding average lower leg angles is shown in Figure 5. The average quasi-static stiffness using this method was 5.81 Nm/rad with a SE of 0.81 Nm/rad.

**Table 3:** **Quasi-static stiffness of the participants’ lower leg from the direct estimation**.

Subject number	Quasi-static stiffness (Nm/rad)
1	8.73
2	5.23
3	5.43
4	3.52
5	3.28
6	5.21
7	4.74
8	3.63
9	7.07
10	11.29
Mean	5.81
SE	0.81

To compare the results of the quasi-static stiffness obtained from the two methods, only the data where the lower leg rotations were within ±0.065 rad was used. The angular motion in the lower leg within the range of ±0.065 rad is near linear when plotted against the estimated torque as shown in Figure 5. In Figure 5, the slope represents the quasi-static stiffness of the lower leg. Since the lower leg crossed the reference angle (0°) 10 times, the experimental data (both angles and torques) were divided into 10 segments of equal length in the range of ±0.065 rad. One-way analysis of variance (ANOVA) was used to compare the results of the quasi-static stiffness from direct experiments, the quasi-static stiffness from the average impedance magnitude in the range of 0–1 Hz, and the stiffness obtained from the transfer function estimated from the impedance measurement. The *p*-value was found to be 0.53 showing that the results obtained from the three methods are statistically similar.

## Discussion

Experimental and analytical methods were presented for the estimation of the mechanical impedance of the human lower leg in the transverse plane. The presented results are bound to the experiment conditions, including suspended lower leg under no load with no muscle contraction with a 90° bent knee. In this configuration, the rotations of the ankle’s talocrural and the subtalar joints are dominant in the recorded dynamics, and any contribution of the tibiofibular joints and knee joint are also measured. Similar to the mechanical impedance of the lower leg in sagittal and frontal planes, the lower leg impedance in the transverse plane showed a behavior close to a second degree function. A second-order mechanical impedance is a function of inertia, visco-elastic properties, and stiffness of the muscles and tendons of the lower leg that contribute to the rotation of the lower leg in the transverse plane. While in higher frequencies, the effects of the inertia are dominant; in the low frequencies, the visco-elastic properties of the lower leg due to the passive characteristics of the soft tissues are dominant. Visco-elastic elements have near constant stiffness below the break frequency, generating the observed near constant impedance magnitude plot at low frequencies. A break frequency would separate high- and low-frequency regions, and its average was determined to be 4.4 ± 0.22 Hz for the populations of this study. The slope of the average magnitude plot was 46.6 ± 1.5 dB/decade that is consistent with the results obtained in DP and IE (Rastgaar et al., 2009; Lee et al., 2014c) with a break frequency of 8–10 Hz. This showed that the break frequency of the impedance function in EI is lower than DP and IE. The average impedance magnitudes in DP and IE directions in the frequency range of less than 2 Hz were reported as 12.61 ± 1.27 and 7.96 ± 0.62 Nm/rad, respectively (Lee et al., 2014c). The quasi-static stiffness in EI direction, from the average impedance magnitude in the range of 0–1 Hz, was 4.90 ± 0.74 Nm/rad, indicating the lower leg is most compliant in the transverse plane.

For the estimated impedance function, the coherence was close to unity (average of 0.92) at all frequencies up to 30 Hz with a minimum of 0.85 at 0.89 Hz, indicating that lower leg impedance in EI is well characterized by linear models under the given experimental conditions. This coherence is similar to the reported coherence for the estimated ankle mechanical impedance function in DP and IE directions (Rastgaar et al., 2009; Ficanha and Rastgaar, 2014; Lee et al., 2014c). Additionally, the analysis method requires that the actuator impedance function to be identified using a similar stochastic identification method and subtracted from the impedance of the combined lower leg and actuator. The coherence for the impedance function for the actuator alone was 0.93, implying a plausible linear behavior of the experiment setup.

Fitting a second-order system to the frequency domain data of each subject’s impedance estimation, a transfer function was obtained. From the second-order model, an average inertia of 4.66 ± 0.70 Nm/rad, average damping of 2.25 ± 0.43 Nm/(rad/s), and an average moment of inertia of 0.37 ± 0.03 kg.m^2^ were obtained. All three parameters show similar variance among the subjects. The average estimated stiffness from the transfer function was 0.24 Nm/rad, which was smaller than the average quasi-static stiffness averaged from the impedance measurement in the range of 0–1 Hz, but it was within its SE limits. The smaller value in the stiffness from the estimated transfer function is expected as the quasi-static stiffness from the impedance measurement takes the effects of damping and inertia within the range of 0–1 Hz into account.

The quasi-static stiffness from direct estimation was 5.81 ± 0.81 Nm/rad compared to 4.9 ± 0.74 Nm/rad for the quasi-static stiffness averaged over the impedance magnitude in the range of 0–1 Hz, and 4.66 Nm/rad ± 0.70 from the estimated transfer function stiffness. The ANOVA was used to compare the results of the three methods. The *p*-value was found to be 0.53, indicating no statistically significant difference between the results.

The presented results can be used on the evaluation of patients suffering from spasticity due to stroke or patients with multiple sclerosis. It can be used to quantify the level of impairment and monitor the progress during the course of treatment. In addition, the results of the study provide a base for furthering our understanding of the lower leg musculoskeletal dynamics. During walking, the lower leg will be under varying load during the stance phase. Additionally, the lower leg’s muscles co-contractions would change the lower leg impedance during the stance phase. The presented experiment and analysis method will be extended to estimate the mechanical impedance of the lower leg with different muscle activation levels, and different knee and ankle angles to define a relationship between muscle activation levels and the mechanical impedance of the lower leg. This may provide design parameters for the development of ankle–foot prostheses with characteristics comparable to the human lower leg. This is particularly important since designing protocols for the estimation of the mechanical impedance of the lower leg or ankle in the transverse plane during walking is challenging. This is evident from the recent studies on estimation of the mechanical impedance of the ankle in the sagittal and frontal planes during stance phase of gait (Lee et al., 2012; Rouse et al., 2014; Ficanha et al., 2015a); however, there has not been any study in the transverse plane. The development of state of the art ankle–foot prostheses may greatly benefit from the estimation of the time-varying mechanical impedance of the human ankle and lower leg in all three anatomical planes, which will be the focus of future work.

## Conclusion

This paper described the protocols and results for the estimation of the dynamic mechanical impedance and the quasi-static stiffness of the human lower leg in the EI rotation direction. Under the conditions of this experiment, the participants’ lower legs were suspended under no load, with knees bent 90°, and relaxed muscles. In the presented configuration, the recorded lower leg dynamics are due to the rotations of the ankle’s talocrural and the subtalar joints, and any contribution of the tibiofibular joints and knee joint. For dynamic mechanical impedance estimation, pseudo-random torque perturbations were applied to the foot, causing its movement in the transverse plane. For the quasi-static stiffness estimation, ramp perturbations with a constant velocity of 0.4 rad/s were applied to the foot, generating the quasi-static motion of the foot in the transverse plane. The dynamic mechanical impedance of the lower leg was estimated in frequency domain with an average coherence of 0.92 within the frequency range of 0–30 Hz, implying a linear correlation between the recorded angular displacement and the torques. The mean magnitude of the stiffness from the averaged impedance of the lower leg in the range of 0–1 Hz was 4.9 ± 0.74 Nm/rad. The results of the experiment for direct estimation of the quasi-static stiffness of the lower leg resulted in the means value of 5.8 ± 0.81 Nm/rad. An ANOVA determined that the difference in the estimated values for the stiffness from the two experiments were not statistically significant.

## Author Contributions

EF contributed in developing the hardware, experiments, data analysis, and writing the material. GR contributed on the experiments and data analysis. MR contributed on the data analysis and writing the material.

## Conflict of Interest Statement

The authors declare that the research was conducted in the absence of any commercial or financial relationships that could be construed as a potential conflict of interest.
